# Differential gene expression in leaves and roots of *Hydrangea serrata* treated with aluminium chloride

**DOI:** 10.3389/fpls.2024.1412189

**Published:** 2024-09-03

**Authors:** Anna-Catharina Scholpp, Hanna Marie Schilbert, Prisca Viehöver, Bernd Weisshaar, Michael Beckstette, Judith Martha Neumann, Hanna Bednarz, Karsten Niehaus

**Affiliations:** ^1^ Metabolome- and Proteome Research, Faculty of Biology & CeBiTec, Bielefeld University, Bielefeld, Germany; ^2^ Genetics and Genomics of Plants, Faculty of Biology, CeBiTec, Bielefeld University, Bielefeld, Germany; ^3^ Computational Metagenomics, Faculty of Technology & CeBiTec, Bielefeld University, Bielefeld, Germany; ^4^ AG 3 Anatomy and Cell Biology, Medical School OWL, Bielefeld, Germany

**Keywords:** *Hydrangea serrata*, aluminium stress, differential transcriptome analysis, secondary metabilites, dihydrocoumarin

## Abstract

*Hydrangea serrata*, also knowen as the Japanese tea hortensia, is known for its sweet taste and health properties of bevarages produced from this plant. The *H. serrata* 3,4-dihydroisocoumarins, hydrangenol and phyllodulcin harbour a variety of biological activities and pharmacological properties. Therefore, a detailed understanding of dihydroisocoumarin biosynthesis in *H. serrata* is of major interest. Their biosynthesis is assumed to be enhanced by elicitors and mediated by polyketide synthases like in cases of phenylpropanoid derived phytoalexins. A *de-novo* transcriptome assembly of leaves and roots from the aluminium chloride treatment group versus the control group alongside with annotation was generated. Secondary plant metabolites were analysed by LC-MS. It revealed that a terpene synthase and a triterpenoid synthase gene as well as lignin biosynthesis encoding genes were upregulated in roots. Many genes for transporters, glycosyl, and other transferases as well as glycosylases were found to be differentially expressed in both organs. As no differentially expressed polyketide synthase gene homolog was found, the relative leaf and root 3,4-dihydroisocoumarin content was analysed by LC-MS measurement. Although *Hydrangea* species are known for their aluminium detoxification using phenylpropanoid-derived compounds, the levels of 3,4- dihydroisocoumarins were not enhanced. In this metabolite analysis, an organ- specific accumulation profile of hydrangenol, phyllodulcin, hydrangeic acid and their mono- and di-glycosides was figured out.

## 1 Introduction

The Japanese mountain Hydrangea (*Hydrangea serrata*) is an important cultural and medical plant due to the content of specialised metabolites. A traditional tea is prepared from the leaves. Health properties of *H. serrata* “Oamacha” and closely related cultivars are mainly due to 3,4-dihydroisocoumarins (DHICs) including hydrangenol (HYD), phyllodulcin (PHY) and stilbene carboxylic acids like hydrangeic acid (Hashimoto et al., 1979; Liu et al., 2013). They share similar positive health effects and anti-microbial properties with the group of isocoumarins (Saddiqa et al., 2017). In contrast to the mostly bitter-tasting isocoumarins, PHY tastes sweet and is traditionally consumed in tea and beverages (Yamato and Hashigaki, 1979). Within the genus *Hydrangea*, *H. serrata* the varieties “Oamacha” and “Amagi Amacha” (classified as a subspecies of *H. macrophylla Thunbergii*) is the only natural sweetener-producing member (Çiçek et al., 2018). The bioconversion of PHY-glycoside to the aglycon as high-intensity sweetener from *Hydrangea macrophylla Thunbergii* leaves is established (Jung et al., 2016).

PHY and HYD occur as glycosides or aglycons and have a structure and synthesis similar to stilbenoids or flavonoids (Asahina and Asano, 1929; Kuć and Rush, 1985; Liu et al., 2013). HYD and PHY are proposed to be derived from hydrangeic acid, a stilbene carboxylic acid (Akiyama et al., 1999; Eckermann et al., 2003; Schröder and Schröder, 1990). Like for other phenylpropanoids, the precursors phenylalanine and tyrosine are derived from the shikimate pathway (D’Auria and Gershenzon, 2005; Herrmann and Weaver, 1999). These aromatic amino acids are converted via phenylpropanoid pathway into *p*-coumarate-CoA (Hahlbrock and Scheel, 1989). An elongation of this intermediate with malonyl-CoA occurs 3× in addition to ring closure and is catalysed by a polyketide synthase (PKS) (Ibrahim and Towers, 1962). A type III PKS subgroup, the CHS family, includes chalcone synthases and stilbene synthases. Together they produce a large group of plant phenylpropanoids including many phytoalexins from specialised metabolism. Phytoalexins play important roles in plant defence, stress responses, or detoxification (Francini et al., 2019; Gundlach et al., 1992; Herrmann and Weaver, 1999). Their biosynthesis can be induced externally by abiotic stressors such as UV radiation, high light, drought, salinity, and toxic compounds such as metals and biotic stressors including certain signal molecules (elicitors) released upon attack by pathogenic organisms or herbivores (Adrian et al., 1996; Baier et al., 1999; Matern, 1991). The phytoalexin spectrum is huge. Terpenoids that are composed of isoprene subunits are non-phenylpropanoid derived phytoalexins (Chen et al., 2019; Ebel, 1986; Kuć and Rush, 1985; Li et al., 2015).

*Hydrangea* species are commonly known for their robustness against aluminium and also for their decorative flowers. *H. serrata* and *H. macrophylla* display a variation of sepal colour in response to soil pH change. The flowers are pink in alkaline and turn blue in acidic soil. Aluminium ions are complexed with two phenylpropanoids. This hydrangea blue complex is deposited in the sepals as means of detoxification (Ito et al., 2019; Schreiber et al., 2011). AlCl_3_ is toxic to plants according to other studies (Rahman and Upadhyaya, 2021). It changes gene expression upon stress response and especially effects the roots (Zhou et al., 2016). The response to treatment with AlCl_3_ on global changes in transcript levels was analysed in *H. macrophylla* which produces no DHICs (Chen et al., 2015). Other phenylpropanoids are inducible by AlCl_3_ or CuCl_2_ treatment (Adrian et al., 1996; Hanawa et al., 1992; Hossain et al., 2023; Schmidlin et al., 2008; Wang et al., 2022). The focus of our study was to observe whether phytoalexins or phenylpropanoids like DHICs in *H. serrata* can be induced by AlCl_3_ treatment. Phytoalexins in hydrangeas were not described. Some monoterpenoids, the iridoids-glycosides from hydrangeas were reviewed by Gousiadou et al. (2016). Secoiridoid glycosides and hydrangeosides A and C were isolated from *H. serrata* leaves (Inouye et al., 1980; Kikuchi et al., 2008).

In this study, we aim to describe changes in the *H. serrata* transcriptome under abiotic stress and compare the effects on roots and leaves. Aluminium chloride-induced alterations in leaf and root transcripts were analyzed to provide insights into specialized metabolism-related gene activity. Utilizing the Illumina NextSeq2000 technique, we employed a deep sequencing approach, which offers a solid foundation for further data analysis and comparisons using the established dataset. The *H. serrata* transcriptome data from this study includes differentially expressed sequences of terpenoid synthases and genes involved in monolignol biosynthesis. This dataset can be used to search for novel genes and gene functions. Additionally, our findings suggest that *H. serrata* is unlikely to produce phenylpropanoids in response to aluminium stress.

## 2 Materials and methods

### 2.1 Plant material

*H. serrata* (*H. macrophylla sups. serrata* “Oamacha”) plants of about 40 cm height (GartenBaumschule TIMM, Reppenstedt, Germany) were cultivated in the greenhouse at the Bielefeld University. The identity of the plants was confirmed by the production of characteristic secondary metabolites (see results) and by the morphology. According to the leaf and flower morphology the analysed species is *H. serrata*. We follow the taxonomical description of the Royal Botanical Garden Kew (https://powo.science.kew.org). Ten individual plants were purchased as one cultivar and divided into two groups. Five plants in the control group were treated daily with 100 mL of tap water for 7 days. The treatment group consisted of the other five plants, which were daily watered with 100 mL of 100 mM AlCl_3_ (Carl Roth, Karlsruhe, Germany) solution for 7 days. The plants were kept in 12 l containers with common potting soil. From every group, a pool sample of vital leaves was collected with *N* = 8 per plant. In parallel, a pool sample of about 2 g vital and rinsed roots were collected per individual plant of every group. All pool samples were immediately frozen in liquid nitrogen and stored at −80°C.

### 2.2 Total RNA extraction

The total RNA was extracted from 95 to 99 mg of frozen sample using the Spectrum Plant Total RNA Kit (Sigma-Aldrich, Taufkirchen, Germany) according to the supplier’s protocol. Sixteen samples were prepared, consisting of four biological replicates for each condition from leaves and roots. On-Column DNase I Digestion Set from Sigma-Aldrich (Taufkirchen, Germany) was used according to the supplier’s instructions. For RNA elution nuclease-free water was used (Merck, Darmstadt, Germany). The RNA quality was validated using a 2,100 Bioanalyzer system (Agilent, Santa Clara, CA, USA). Samples with RNA integrity number above 8 went into library construction.

### 2.3 Library construction and cDNA sequencing

Sequencing libraries were constructed from 3 µg of total RNA, following the TrueSeq v2 protocol (Illumina, Cambridge, UK). Paired-end sequencing was performed on an Illumina NextSeq2000 instrument at the Sequencing Core Facility of the Center for Biotechnology (CeBiTec) at Bielefeld University. A total of 24 samples libraries were applied on two lanes of a P3 flow cell. The paired-end read length was 150 bp and the total amount was 1.3 Mrd reads per sample.

### 2.4 Data processing and *de-novo* transcriptome assembly using Trinity

Illumina paired-end reads were processed analogous to the best practises guideline for a *de-novo* assembly using Trinity (Freedman and Weeks, 2020; Grabherr et al., 2011). All computational operations were calculated using the de.NBI cloud (https://www.denbi.de/cloud). First, read quality was examined by FastQC v0.11.3 (Andrews, 2010). Removal of erroneous k-mers was made by applying rCorrector v1.0.4 (Song and Florea, 2015). Adapter and low-quality regions were trimmed with TrimGalore! v0.6.6 (Krueger, 2015). Trimmed reads were obtained and a total number of 424,302,023 bases was assembled using Trinity v2.12.0 in genome independent- and paired-end mode, including normalisation to targeted maximum achievable coverage.

### 2.5 Functional annotation and differential expression analysis

The transcriptome assembly quality was rated via (BUSCO v3.0.2) (Simão et al., 2015) using the reference dataset of embryophyte_odb9 containing 1,440 Benchmarking Universal Single-Copy Orthologs (BUSCOs) of 30 species. TransDecoder (Haas et al., 2013) was used to identify open reading frames (ORFs) and translate ORFs to peptide sequences with a minimum length of 100 amino acids. Genes were annotated by transferring the *A. thaliana* Araport11 (Cheng et al., 2016) functional annotation to the *H. serrata* gene models as described before (Schilbert et al., 2021). The quantitative transcriptome data were obtained from read counts per contiq. Therefore, Kallisto v.0.44 (Bray et al., 2016) was applied using default parameters to quantify transcript abundances and to generate count tables. The generated count tables were subjected to differential gene expression analysis via DESeq2 to obtain the log_2_ fold change for each gene (Love et al., 2014). Differentially expressed genes (DEGs) with a log_2_ fold change >2 and adjusted *p*-value <0.1 between leaves versus leaves_ AlCl_3_ and roots versus roots_ AlCl_3_ were identified. Identified DEGs were visualized by MapMan as follows: The functional annotations were sorted by biological categories of the proteins using Mercator4 v2.0 (Schwacke et al., 2019) and mapped together with the quantitative transcriptome data onto specialised metabolite pathways in MapMan 3.5.1R2 (Usadel et al., 2009). Pathway map files were adjusted to the biological categories using GIMP 2.10.18. The DEGs were displayed colour scaled on pathway maps and summarized in tables (**Supplementary Material**). The biological context was obtained from protein category bins using Mercator4 and MapMan. DEGs from the selected biological categories of interest were visualised in Venn diagrams using http://bioinformatics.psb.ugent.be/software.

### 2.6 UHPLC-ESI-QToF-MS analysis for DHIC profiling

Metabolite profiles were measured by UHPLC UltiMate 3000 and DAD (Thermo Scientific, Waltham, Massachusetts, USA) in combination with ESI Bruker MicroToFQ (Bruker Daltonics, Bremen, Germany) in negative ion mode. An Eurosphere II 100 mm × 2 mm C_18_ column (Knauer, Berlin Germany) was used for the separation in chromatography at a flow rate of 0.45 mL/min. The mobile phase was set to a gradient starting with 10% acetonitrile including 0.1% formic acid (B) and 90% water including 0.1% formic acid (A) for 0–5 min. Followed by 10%–25% B 5–10 min, 25%–45% B 10–20 min, 45%–65% B 20–30 min, 65%–100% B 30–40 min, hold 40–50 min, 100%–10% B 50–55 min. The wavelengths were set to 340 and 280 nm. HPLC grade water, solvents, and formic acid were purchased from TH Geyer (Höxter-Stahle, Germany). The technical settings were taken and modified from flavonoid-glycoside detection by Lin & Harnly (2007). Negative ions of sodium formate (VWR Chemicals, Haasrode, Belgium) was used for initial mass calibration in every first 60 s of sample acquisition. Mass spectra were taken for a range of 120–1000 m/z. Naringenin chalcone (Carl Roth, Karlsruhe, Germany) was used as internal standard. For mass identification of PHY and HYD a “Sweet Hydrangea Leaf Dihydroisocoumarin” reference was measured (FUJIFILM Wako Chemicals Europe, Neuss, Germany).

## 3 Results

*H. serrata* plants of about 40 cm height were grown in potting soil under optimal greenhouse conditions. The plants were treated with AlCl_3_ solution or with tap water as control. Leaves and roots were harvested, RNA was extracted and cDNA libraries were sequenced using the Illumina technology. The number of resulting 580,770 contigs includes isoforms, allelic variants and other sequences. The application of BUSCO revealed 96.2% complete BUSCO genes in the *de-novo H. serrata* transcriptome assembly. Among the complete BUSCOs, 10.4% were single-copy genes whereas 85.8% were duplicated. Moreover, 1.6% of all BUSCO genes were fragmented and only 2.2% were missing.

**Figure 1:**
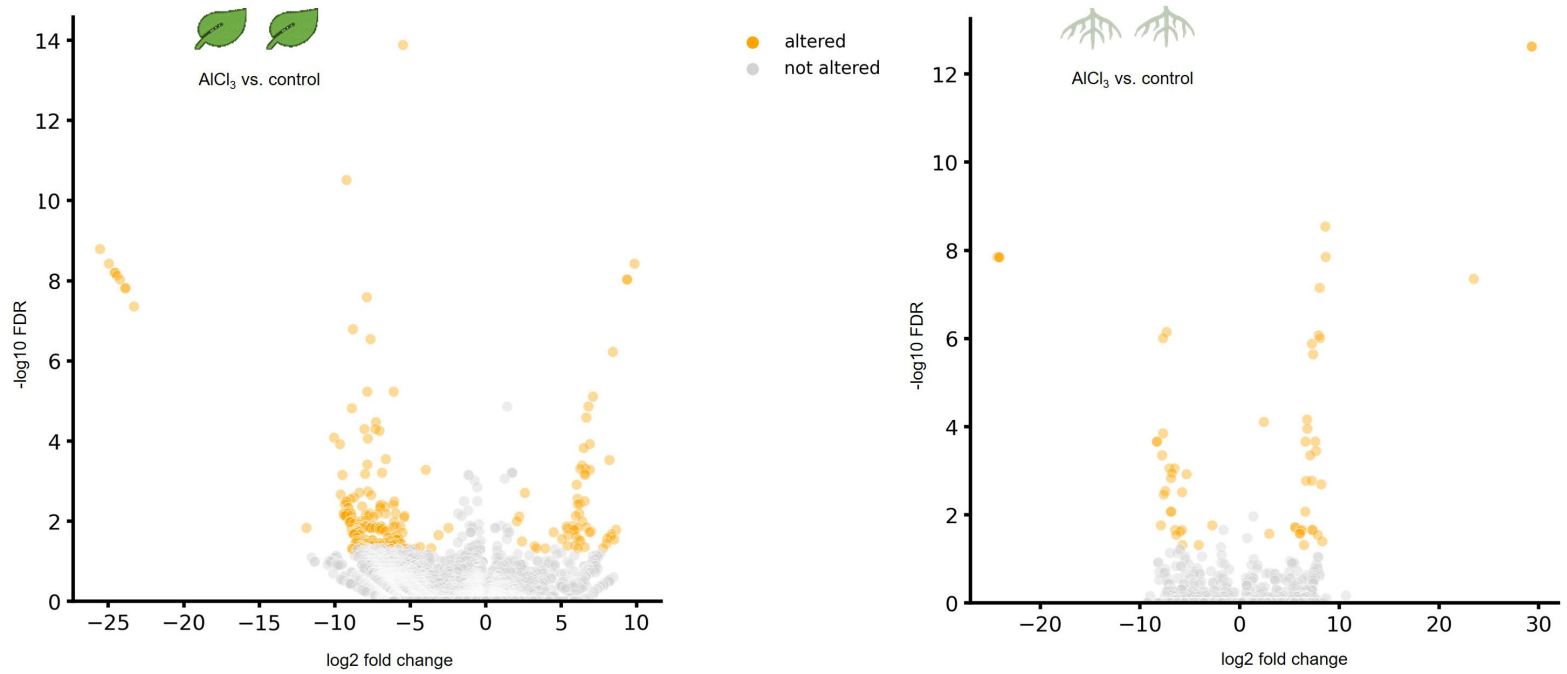
Volcano plots showing differentially expressed genes (DEGs) in leaves treated with aluminium chloride (AlCl_3_) compared to control leaves as well as in AlCl_3_-treated roots compared to control roots. DEGs with a log_2_ fold chance >2 and <−2 and an adjusted *p*-value of <0.05 are marked in orange. FDR is the false discovery rate of the adjusted *p*-value. The individual *H*. *serrata* plants were treated with tap water in the control group and with 100 mM AlCl_3_-solution in the treatment group for 5 days. Each condition group consists of four biological replicates.

A differential expression analysis between AlCl_3_-treated organs and corresponding controls was made using DESeq. As displayed in volcano plots, a higher number of genes were differentially expressed in AlCl_3_-treated leaves compared to AlCl_3_-treated roots (**Figure 1**).

A total of 119,756 representative peptide sequences were annotated via reciprocal best BLAST hits (RBHs) and best BLAST hits (BBHs) using *Arabidopsis thaliana* data. A total of 75,100 annotated sequences were sorted in biological categories plus 42,432 enzyme classification categorized in more than 50 bins using Mercator 4.0. Each bin contained second and third order subclasses or more for detailed specification.

### 3.1 Biological categorisation of differential expressed genes in response to aluminium chloride treatment

**Figure 2:**
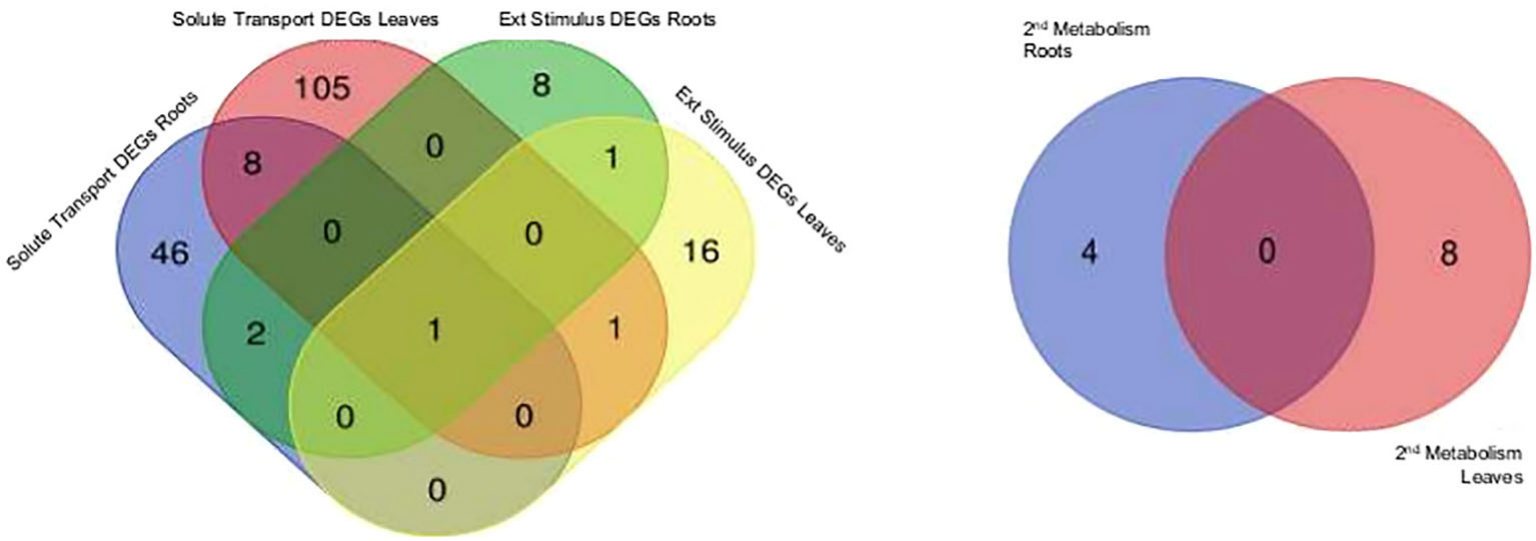
Differentially expressed genes (DEGs) are displayed for selected biologicals categories based on Mercator4 protein assignment to functional context. External stimuli response and solute transport in roots treated with aluminium chloride (AlCl_3_) compared to control roots as well as in leaves treated with AlCl_3_ compared to control leaves and specialised metabolism in roots treated with AlCl_3_ compared to control roots and in leaves treated with AlCl_3_ compared to control leaves. Both Venn diagrams show tissue specific DEG amounts, log_2_ fold change >2; <−2 and an adjusted *p*-value <0.1 from aluminium chloride (AlCl_3_) treated roots and leaves compared to the respective control. DEGs with same expression level are in the overlap.

**Figure 3:**
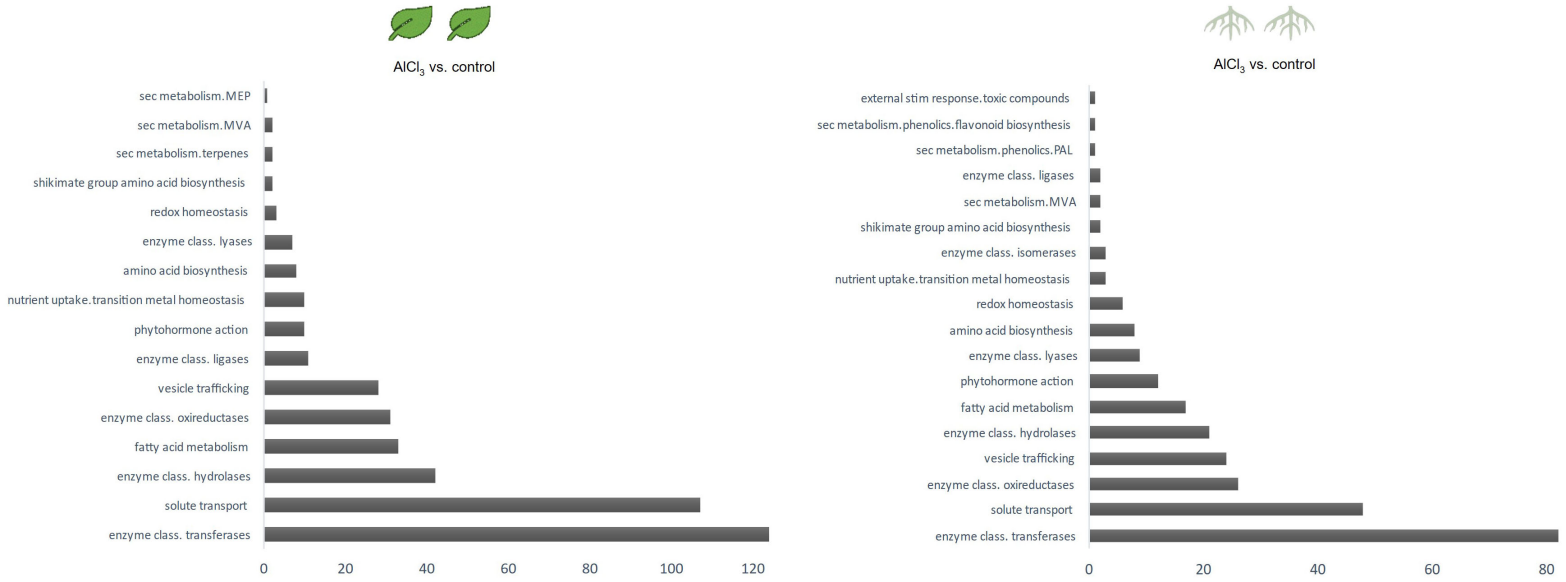
Counts of differentially expressed genes per biological category in leaves treated with aluminium chloride (AlCl_3_) compared to control leaves and in roots treated with AlCl_3_ compared to control roots. Functional annotated proteins were sorted in biologicals categories using Mercator4. The assigned functional protein categories were selected for specialised metabolism and the corresponding amount of DEGs having a log_2_ fold change >2; <−2 and adjusted *p*-value <0.1 was counted.

Categories of functional annotations were summarized using Mercator4. **Tables 1**, **2** show the strongest regulated genes, filtered for a log_2_ change <4>, and their annotations. Both *H. serrata* tissues showed transcriptional changes in the biological categories of solute transport, phytohormone action, external stimuli response, and specialised metabolism (**Figures 2**, **3**). The most annotations were categorized into enzyme classification and solute transport followed by protein modification and vesicle trafficking (**Figure 2**). Many genes encoding for glycosyltransferases and oxidoreductases were regulated in both tissues. A higher number of glycosylases encoding genes were regulated in the roots (**Figure 2**; **Tables 1**, **2**).

**Table 1:** Functional annotations of differential expressed genes from AlCl_3_-treated H. serrata roots versus control with a log_2_ fold change.

Functional annotation	Log_2_ fold change
Lazarus1 (LAZ1, a DUF300 protein) homologous transmembrane protein	4.1
Terpene synthase	4.1
Glycosylases (six cases)	4.1; 5.4; 5.7; 5.9; 6.1; 6.4
Oxidoreductases (six cases)	4.2; 4.8; 5.1; 5.6; 6.4; 7.7;
ABCG transporter	4.6
7-deoxyloganic acid hydroxylase cytochrome p450 cyp72a219 (not further assigned)	5.2
Strigolactone signal modulator (SMXL) regulator protein from phytohormone action	5.4
TNL-mediated regulator protein from external stimuli response	5.5
calcium-dependent anion channel (Ca-ClC)	5.5
MYB domain containing proteins (three cases)	5.5; 6; 6.5
methylsterol monooxygenase from phytosterol metabolism	5.6
P1B-type heavy metal cation-transporting ATPase (HMA)	5.6
acyltransferase	5.9
Triterpenoid synthase	6.1
Glycosyltransferases (two cases)	6.1; 7.2
Carrier-mediated transport.DMT superfamily.solute transporter (NIPA) magnesium transporter	6.8
ABCB transporter (two cases)	7.4; 6.5
Carrier-mediated transport.APC superfamily.APC family.gamma-aminobutyric acid transporter (GABP)	7.9
Glycosylases, (five cases)	−4; −5; −5; −6.1; −7.1
Iron cation sensor protein, iron cation sensor protein (HRZ/BRUTUS)	−5.3
DMT superfamily.solute transporter (NIPA), magnesium transporter	−5.3
Hexosyltransferases, (two cases)	−5.3; −5.9
Nicotinate transporter (NiaP)	−5.4
Proton:sodium cation antiporter (NHX)	−5.5
Rapid alkalinization factor (RALF) precursor peptide from phytohormone action	−5.5
Glycosyltransferase	−5.9; −5.4
ABC superfamily.ABC1 family.subfamily ABCB transporter	−6.1
Monosaccharide transporter (AZT)	−6.6
Neoxanthin cleavage dioxygenase	−6.7
P4-type ATPase component (ALA)	−6.8
APC superfamily.AAAP family.solute transporter (AAAP)	−6.9
Cytokinin perception and signal transduction.B-type ARR response activator	−6.9
Ferric-citric complex transporter	−7.2
Metal-citrate complex transporter (FRD)	−7.2
Fatty acid metabolism. polymeric acetyl-CoA carboxylase complex.carboxyltransferase subunit alpha	−8.1

**Table 2:** Functional annotations of differential expressed genes from AlCl_3_-treated *H. serrata* leaves versus control with a log_2_ fold change.

Functional annotation	Log_2_ fold change
P4 family.phospholipid flippase complex.P4-type ATPase component (ALA)	4.8
Glycosylase	4.8
Ketoacyl-ACP synthase (mtKAS)	5.6
Phosphatidylinositol biosynthesis.bifunctional inositol pyrophosphate kinase and phosphatase (VIP)	5.7
Clathrin coated vesicle (CCV) machinery.AP-3 cargo adaptor complex.medium subunit mu (AP3M)	5.7
Glycosyltransferase	5.7
Maltose transporter (MEX)	5.8
Acyl-CoA-binding protein (ACBP1/2/3)	6.4
MFS superfamily. UMF23-type solute transporter	6.4
APC superfamily.AAAP family.amino acid transporter (AAP)	6.5
P1B-type heavy metal cation-transporting ATPase (HMA)	6.6
CPA-1 family.proton:cation antiporter (NHX)	6.7
Toxic compounds.arsenic.vacuolar sequestration.glutathione S-conjugate transporter	6.7
Hexosyltransferase	6.9
APC superfamily.NCS-2 family.solute transporter (NAT)	7.1
Salicylic acid–binding protein	7.2
Exocytic trafficking.Exocyst complex.component (EXO70)	8.2
Transmembrane protein (not further assigned), (2cases)	5.8; 7.1
Oxidoreductases (four cases)	4.2; 5.7; 6.; 8.3
phb domain containing protein (not further assigned)	−4.2
Glycosylase	−4.5
Caffeoyl-CoA O-methyltransferase	−4.6
Spermidine biosynthesis.S-adenosyl methionine decarboxylase	−6.
Proposed aminodeoxychorismate synthase or anthranilate synthase component (tetrahydrofolate metaboilism)	−6.4
Proposed farnesoic acid O-methyltransferase or loganic acid O-methyltransferase (nut further assigned)	−6.5
Common symbiotic signalling pathway (CSSP), calcium cation channel (DMI1/Pollux/Castor)	−6.5
Channels, VIC superfamily.cation channel (DMI1)	−6.5
ABC superfamily, ABC1 family.subfamily, ABCC transporter	−6.7
Carrier-mediated transport.solute transporter (MTCC)	−6.7
Regulatory protein (SCAMP) of exocytic trafficking & prot-scriber: secretory carrier associated	−6.9
Reactive oxygen (ROS)–induced xanthine dehydrogenase	−7.3
Oxidoreductases (four cases)	−6.5; −6.8; −7; −7.3

Among DEGs from AlCl_3_-treated roots (roots_AlCl_3_) versus control with a log_2_ fold change >4, several genes encode for enzymes such as oxidoreductases (six cases), glycosyltransferases (two cases), glycosylases (six cases), and one methylsterol monooxygenase from phytosterol metabolism. Additionally, differentially expressed sequences specialised metabolism include genes encoding terpene synthase and triterpenoid synthase. Other genes were annotated as transporter as well as ion channels.

Downregulated genes with log_2_ fold change <−4 were annotated as glycosylases (five cases), one glycosyltransferase, hexosyltransferases (two cases), and acyltransferases (two cases). Furthermore, downregulated genes encoded for iron cation sensor protein as well as many transporters. A polymeric acetyl-CoA carboxyltransferase subunit from the fatty acid metabolism encoding transcript was significantly downregulated (**Table 1**).

Genes for aluminium or general heavy metal ion response mechanisms like transporters were similarly differentially expressed, like Chen et al., 2015 reported for *H. macrophylla*. One interesting exception is the aluminium-activated malate transporter (QUAC/ALMT) which was slightly downregulated in roots (**Supplementary Material**). Also, in AlCl-treated roots of *H. serrata* the iron cation sensor protein HRZ/BRUTUS, ferric-citric complex transporter, and metal-citrate complex transporter FRD were downregulated.

DEGs in AlCl_3_-treated leaves (leaves_AlCl_3_) versus control with log_2_ fold change >4 was functionally annotated as transporters, transmembrane proteins (two cases), one acetyl-CoA binding protein, one salicylic acid binding protein, one glycosylase, oxidoreductases (three cases), one hexosyltransferase and one glycosyltransferase (**Table 2**).

Downregulated genes with log_2_ fold change <−4 encoded for other transporters, calcium cation channel, oxidoreductases (four cases), one glycosylase, and one ROS-induced xanthine dehydrogenase (**Table 2**).

### 3.2 Specialised metabolism-specific differential gene expression in response to aluminium chloride treatment

**Figure 4:**
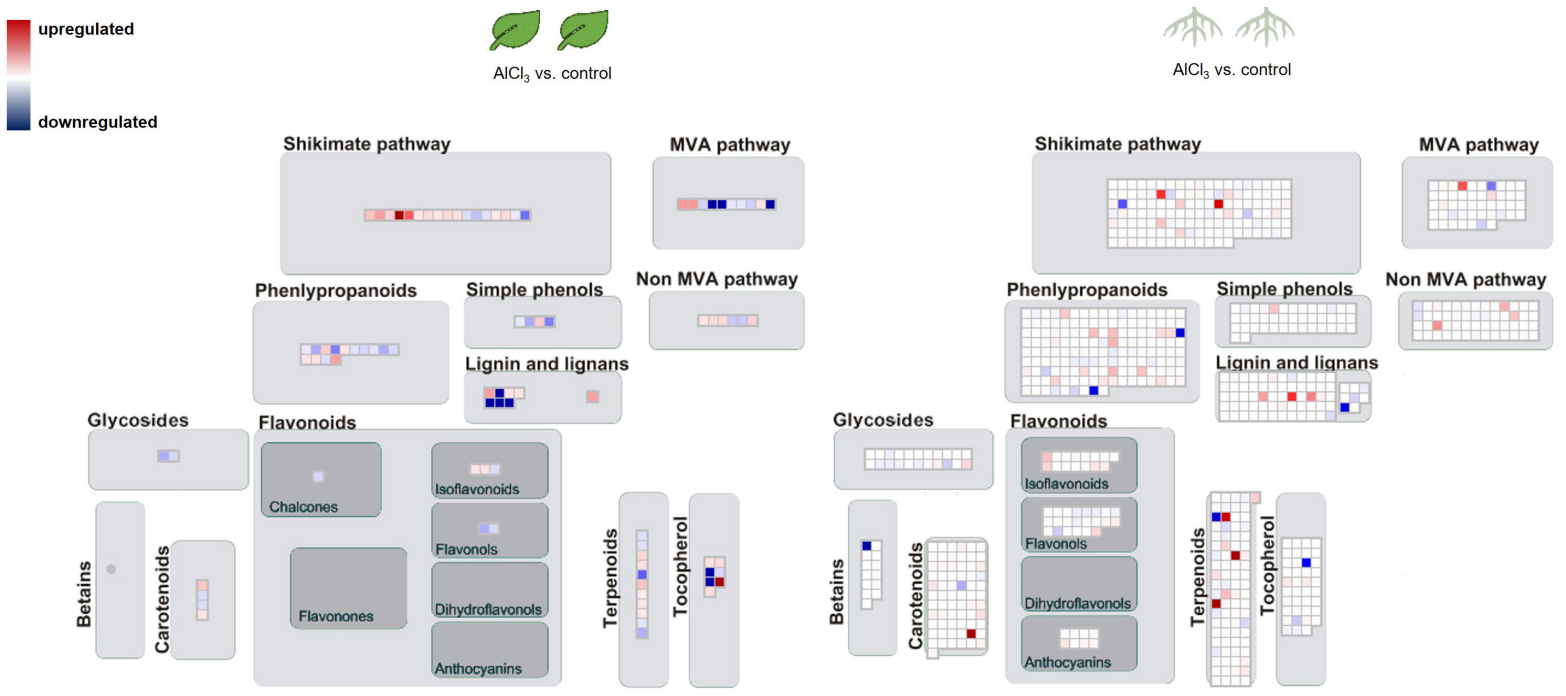
Pathway map for *H*. *serrata* specialised metabolism and differential expressed genes (DEGs) from roots treated with aluminium chloride (AlCl_3_) compared to control roots and from leaves treated with AlCl_3_ compared to control leaves. DEGs are shown as coloured squares in a MapMan-based image. Each square stands for one DEG or isoform. The genes were mapped on the pathway image based on biological categories of the encoded proteins which were summarized using Mercator4. The false colour scale represents log_2_ fold change >0 in red and <−0 in blue for adjusted *p*-value <1. Each condition group consists of four biological replicates from *H*. *serrata*. The plants were treated with tap water in the control group and with 100 mM AlCl_3_-solution in the treatment group for 5 days.

**Figure 5:**
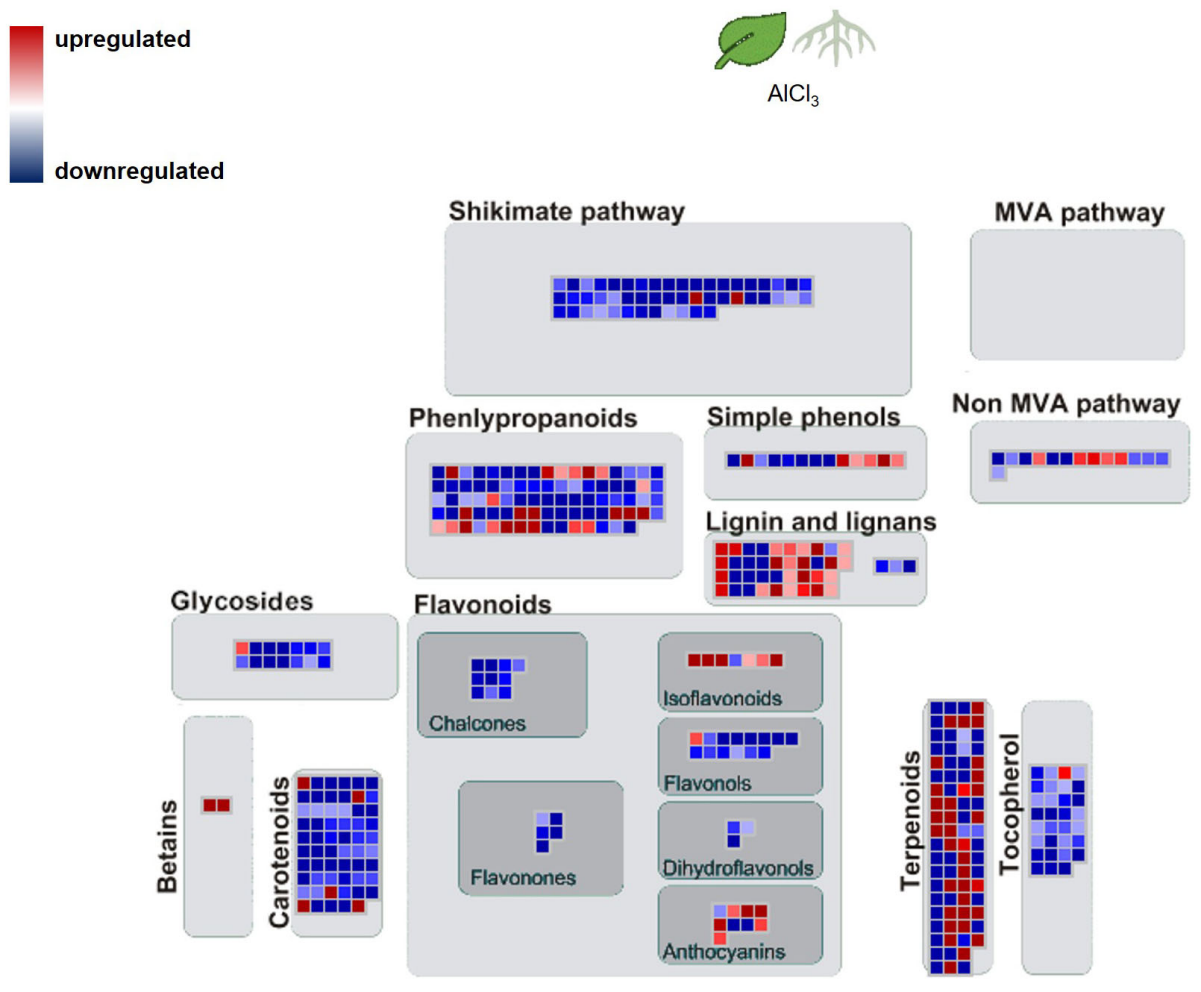
Pathway map of *H. serrata* specialised metabolism and differential expressed genes (DEGs) from and roots compared to leaves aluminium chloride (AlCl_3_) treatment group. Coloured squares are shown in MapMan-based image. Each square stands for one differentially regulated gene or isoform. The genes were mapped on the pathway image based on biological categories of the encoded proteins which were summarized using Mercator4. The false colour scale represents log_2_ fold change >2 in red and <−2 in blue for *p*-value adjusted <0.05.

The biological categorisation of functional annotated DEGs were mapped onto pathways of the specialised metabolism. These MapMan images show which gene sets were regulated under the presence of AlCl_3_. The most significantly regulated gene sets covered the shikimate and MVA pathway, terpenoid, lignan, and lignin biosyntheses (**Figures 4**, **5**). More of these genes were upregulated in roots_AlCl_3_ than in leaves_AlCl_3_ (**Figure 4**). One PAL isoform was downregulated in the roots (−2). Additionally, in the leaves an isoprenyl diphosphate synthase (−3.2) was downregulated, a triterpenoid synthase (6.1), a terpene synthase (4.1), and an MEP pathway reductase (2.3) were upregulated. Obviously, AlCl_3_ induced stress activated genes of the terpenoid pathway and genes encoding for enzymes of the lignin biosynthesis.

**Figure 6:**
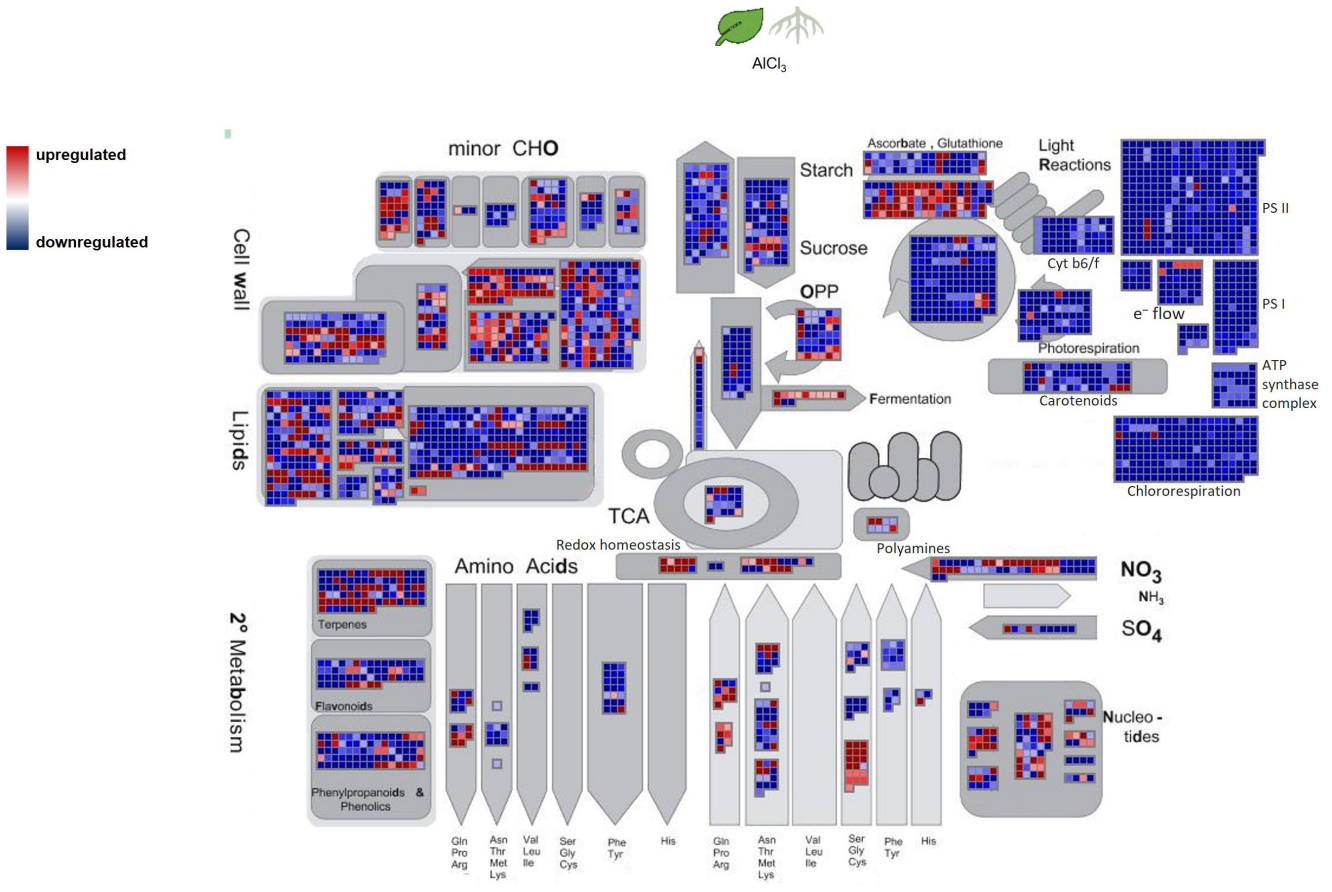
Overview of *H. serrata* metabolism and its involved differentially expressed genes (DEGs) from roots compared to leaves. DEGs are shown as coloured squares in a MapMan-based image. Each square stands for one DEG or isoform. The genes were mapped on the pathway image based on biological categories of the encoded proteins which were summarized using Mercator4. The. The false colour scale represents log_2_ fold change >2 in red and <−2 in blue for *p*-value adjusted <0.05.

The MapMan image (**Figure 6**) depicts a larger overview of the genes which were regulated in roots_AlCl_3_ compared to leaves_AlCl_3_. Differential expression with log_2_ fold change <−2 and >2 and adjusted *p*-value <0.05 occurred in gene sets related to metabolism of terpenes, flavonoids, and other phenylpropanoids. Many shikimate, MVA pathway, carotenoid, tocopherol, and phenylpropanoid synthesis genes were downregulated, same as in the flavanone, flavanol, dihydroflavonol, glycoside, and for some genes in chalcone, tocopherol, and carotenoid syntheses. Betain, a few carotenoid, terpenoid, lignin, one lignan, isoflavonoid, and anthocyanin synthesis-related genes were upregulated (**Figure 5**). Two interesting genes were upregulated. They encode for one lignin, monolignol biosynthesis involved caffeoyl-CoA 3-O-methyltransferase (2.3) and one (CCoA-OMT) terpenoid biosynthesis, mevalonate pathway involved acetyl-CoA C-acyltransferase (2.2).

### 3.3 LC-MS analysis of organ specific dihydroisocoumarin levels

The Illumina NextSeq2000 cDNA sequencing was followed by a general transcriptome analysis. In DHIC biosynthesis or among PKS homologues, no differential gene expression was observed. It was the aim of this study to decipher differences on DHIC level.

No significant alteration as a response to aluminium chloride treatment, in terms of DHICs was detected, in the LC-MS analysis of the methanolic extracts. The metabolite analysis shows an organ specific accumulation profile of DHICs and mono- as well as di-glycosidic versions (**Table 3**). Also, a small elicitor study using yeast extract solution on *H. serrata* leaves, as well as a drought experiment and jasmonic acid treatment neither enhanced HYD nor PHY in the LC-MS measurement of leaf or root extracts (data not shown). Interestingly, the plants showed signs of stress response in specialised metabolites. One sign was the accumulation of anthocyanins, which was visible to the eye in the experiment that combined high light and drought. Another observation was the formation of necrosis in leaves treated with yeast-elicitor solution (data not shown). The DHIC content was not affected in the described experiments (data not shown).

**Table 3:** Relative content of dihydroisocoumarins (DHICs) in extracts from aluminium chloride (AlCl_3_) treated and control *Hydrangea serrata* leaves and roots.

Mass peak [m/z]	Compound name	Leaves contr.	Leaves AlCl_3_	Roots contr.	Roots AlCl_3_	Leaves contr.	Leaves AlCl_3_	Roots contr.	Roots AlCl_3_
		Rel. content [a.u]				Standard deviation			
589.2	H-2glyc	0.45	0.41	0.64	1.07	0.10	0.08	0.12	0.22
627.19	P-2glyc	0.54	0.90	0.29	0.40	0.19	0.11	0.08	0.10
417.16	H-glyc	0.21	0.22	0.64	0.86	0.04	0.01	0.09	0.25
447.17	P-glyc	0.63	0.72	0.01	0.02	0.07	0.09	0.00	0.01
255.09	H Acid*	0.02	0.04	0.40	0.37	0.03	0.03	0.17	0.08
255.09	H-aglyc	0.52	0.51	1.97	1.82	0.27	0.31	0.20	0.43
285.10	P-aglyc	2.62	2.59	0.03	0.04	1.20	1.06	0.01	0.00
		Retention time [min]							
		17.3		15.7					
		19.5		16.4					
		22.5		20.1					
		23.4		21.1					
		29.2		26.5					
		32.3		28.8					
		33.5		30.1					

*Not confirmed by standard.

DHIC aglycons (H-aglyc and P-aglyc), mono- (H-glyc, P-glyc) and di-glycosides (H-2glyc, P-2glyc) occur in specific contents which differ between leaves and roots. Hydrangenol (H) and one hydrangenol isobar, putatively hydrangeic acid (H Acid), were more abundant in roots. The content of phyllodulcin (P-aglyc and P-glyc) was high in leaves but very low in roots. Both di-glycosides (H-2glyc, P-2glyc) were detected in leaves as well as in roots. Four biological replicates were analysed for each condition and organ. The plants were treated with tap water in the control group and with 100 mM AlCl_3_-solution in the treatment group for 5 days.

UHPLC-ESI-QToF-MS measurement was performed after methanolic DHIC extraction.

## 4 Discussion

Illumina NextSeq 2000 sequencing of cDNAs is a powerful technique to gain insight into differences in gene expression related to plant specialised metabolism under abiotic stress. This study is based on paired-end reads which were sequenced in two technical replicates from sixteen cDNA libraries, consisting of four biological replicates for each condition applied on two *H. serrata* organs. A total of 380,000 different transcripts was proposed as genes by Trinity out of 580,770 assembled contigs from *H. serrata* leaf and root sequencing data. For roots from *H. macrophylla* 256,127 unigenes of 401,215 contigs were reported (Chen et al., 2015). This transcriptome study examined two tissues under aluminium influence and we saw that a higher number of genes were expressed in leaves than in roots. AlCl_3_, in some studies, has been reported to be toxic to plants (Rahman and Upadhyaya, 2021). It changed gene expression as a stress response and especially effected the roots (Zhou et al., 2016). A response of *H. serrata* under AlCl_3_ treatment detected as DEGs was found in roots and additionally in leaves. Similar genes encoding transporter proteins which were found in *H. serrata* were described for *H. macrophylla* roots and studies on other plants (Chen et al., 2015; Wu et al., 2023; Zhang et al., 2019; Zhou et al., 2016).

The transcriptional changes under aluminium chloride treatment were visualized using the MapMan software. Combined with the high-quality functional annotation via data from *A. thaliana* and a Mercator4 summarization, the DEGs were categorized according to the biological context of their functional annotation. Signs of DHIC biosynthesis were not visible in differential gene expression against our expectation. In contrast, genes encoding for enzymes of the terpenoid biosynthesis and cell wall lignification were upregulated.

From the context of specialised metabolism many genes were downregulated in roots and only a few were upregulated related to isoflavonol, anthocyanin, carotene, and betaine synthesis. Additionally, lignin- and lignan biosynthesis related genes were upregulated in both tissues. Enhanced lignin synthesis is known as a part of stress defence in plants (Moura et al., 2010; Sasaki et al., 1996; Vanholme et al., 2012). Metal ions cause oxidative stress as ROS in plants. The data show evidence that ROS detoxifying mechanisms were activated or enhanced. Genes from the sectors of glutathione synthesis, sulfur containing amino acids, and redox homeostasis were upregulated in roots, but not in leaves.

Tissue-specific alterations in roots_ AlCl_3_ versus control roots showed that genes encoding for genes related to betain, tocopherol, and phenylpropanoid metabolisms were downregulated. Genes related to MVA, MEP, non-MVA, carotenoid, and shikimate pathway were upregulated. Phenylpropanoid synthesis-involved genes were less expressed than expected. It is remarkable that no homologue of Type III PKS was among DEGs under AlCl_3_ treatment. Moreover, genes from terpenoid and lignin metabolism were upregulated. Upregulated DEGs from leaves_ AlCl_3_ versus control leaves belong to MVA, shikimate, tocopherol, and minor to phenylpropanoid pathways. In addition to various phenylpropanoids, the terpenoids are known to have phytoalexin functions (Chen et al., 2019; Li et al., 2015).

The external stimulation for *H. serrata* DHICs seems difficult because little is known about the biological purpose in the plant. Studies on highlight or shading experiments did not effectuate a change in the DHIC content in leaves (Moll et al., 2021). Also, herbivore attacks did not enhance the DHIC content (Ujihara et al., 1995). Aluminium chloride is another stressor that did not affect the DHIC biosynthesis. A recent study shows that neither ethylene nor salicylic acid had an influence on the HYD and PHY accumulation in *H. serrata* leaves. The HYD and PHY contents in leaves were enhanced due to a methyl jasmonic acid treatment (Preusche et al., 2022). So, methyl jasmonic acid seems to be the right elicitor for the induction of the DHIC biosynthesis and it needs further investigation in the discovery of genes involved in the DHIC biosynthesis and the biological assignment of DHICs.

Important bioactive terpenoids were isolated from hydrangeas, but they were not investigated as phytoalexins. The isolated terpenoids from *H. macrophylla* were identified as secoiridoid glycosides, loganin, secologanin, secologanic acid, and sweroside (Gousiadou et al., 2016; Yoshikawa et al., 1994). Secoiridoid glycosides and hydrangeosides A and C were isolated from *H. serrata* leaves (Inouye et al., 1980; Kikuchi et al., 2008). Other terpenoids then described in literature may exist as inducible phytoalexins. The use of terpenoids as phytoalexins in response to aluminium chloride in *H. serrata* would explain that less transcriptional changes related to phenylpropanoid metabolism were detected in this study. Even this extensive transcriptome dataset for this study on *H. serrata* was generated, its analysis in terms of DHIC was limited. The most limiting factor is the rareness of 3,4-DHICs in *Hydrangea* species and in plant kingdom at all and therefore a lack of suitable reference enzymes in forms of annotated PKS.

## 5 Conclusion

Many aspects about the main mechanisms of aluminium chloride detoxification in hydrangea which were described in other studies were observed in this study as well. It is important to mention that transporter and cell wall stability related genes were differentially expressed as a similar study on *H. macrophylla* showed. This work was more focussed on the specialised metabolites and especially in DHICs. Interestingly, *H. serrata* in contrast to *H. macrophylla* showed changes in the terpenoid biosynthesis as response to aluminium chloride. In terms of DHIC biosynthesis little references on genetic level are available to fully explore the differential gene expression.

## Data Availability

The original contributions presented in the study are publicly available. This data can be found at the National Center for Biotechnology Information (NCBI) using accession number PRJNA1061562.
