# Supplementary material for: Differential gene expression in leaves and roots of Hydrangea serrata treated with aluminium chloride
Source: Front Plant Sci. 2024 Sep 3;15:1412189. doi: 10.3389/fpls.2024.1412189 (PMC11405211; doi:10.3389/fpls.2024.1412189)
Supplement: Supplementary file 4 [file DataSheet1.zip › FiguresSupplementRevised.pdf/FiguresSupplementRevisedTable1.pdf]

Anna-Catharina Scholpp - Supplement Table 1.

**LC parameters**	
solvents	**A** HPLC grade Water + 0.1 % formate
	**B** HPLC grade Acetonitrile + 0.1 % formate
flow	0.45 mL/min
column oven temperature	40 °C
injection volume	10 µL

**ESI-MS parameters**	
dry gas	8 L/min
nebulizer gas	3 Bar
dry temperature	180 °C
mass detection range	120-1000 m/z
collision cell collision energy	40 eV
