# Supplementary figures and images for: Differential gene expression in leaves and roots of Hydrangea serrata treated with aluminium chloride
Source: Front Plant Sci. 2024 Sep 3;15:1412189. doi: 10.3389/fpls.2024.1412189 (PMC11405211; doi:10.3389/fpls.2024.1412189)

Anna-Catharina Scholpp - Supplement Figure 1.

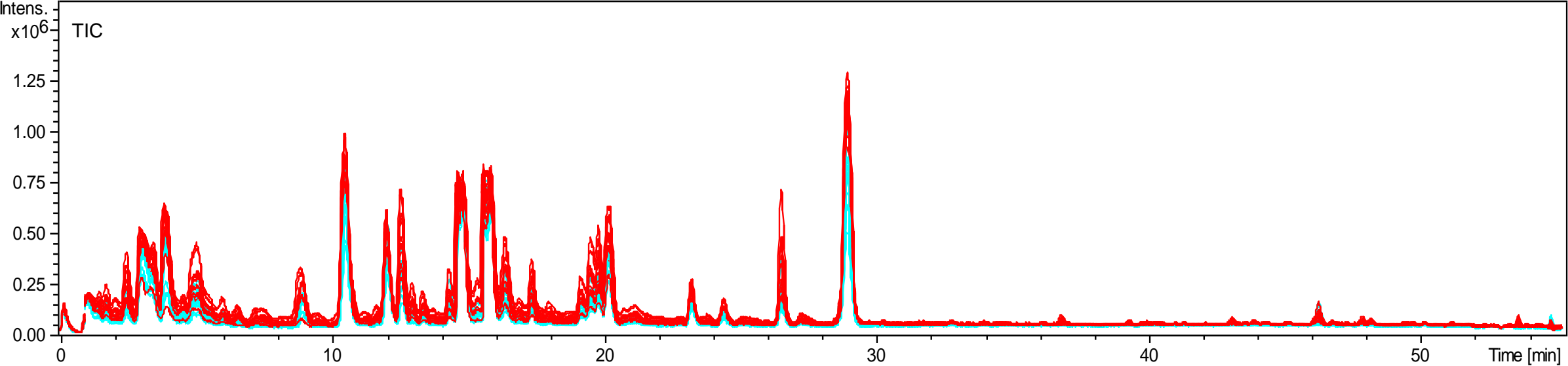

Supplement: Supplementary file 4 [file DataSheet1.zip › FiguresSupplementRevised.pdf/FiguresSupplementFigure1.pdf]

Anna-Catharina Scholpp - Supplement Figure 2.

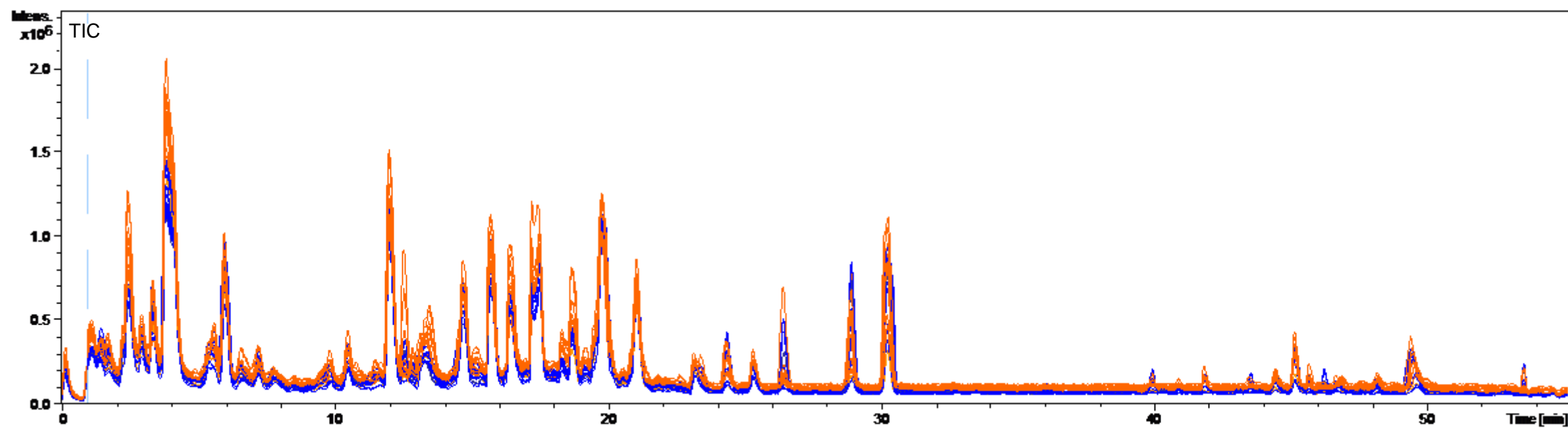

Supplement: Supplementary file 4 [file DataSheet1.zip › FiguresSupplementRevised.pdf/FiguresSupplementRevisedFigure2.pdf]

Anna-Catharina Scholpp - Supplement Figure 3.

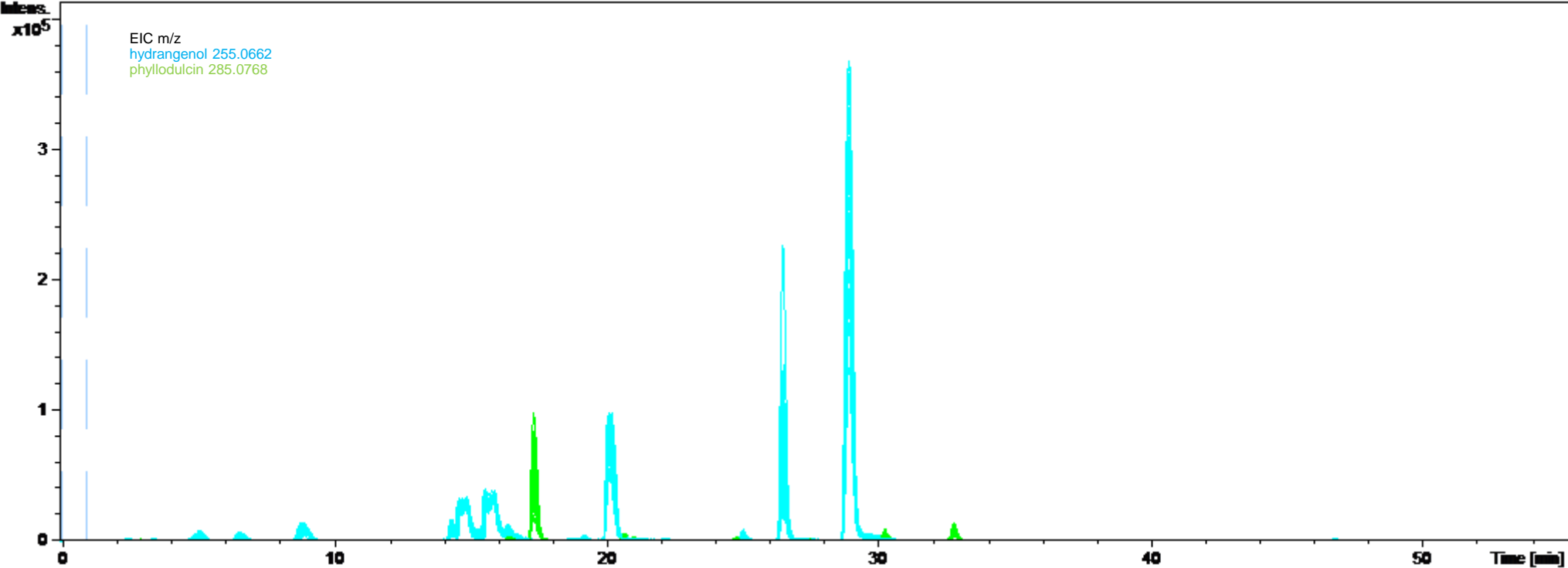

Supplement: Supplementary file 4 [file DataSheet1.zip › FiguresSupplementRevised.pdf/FiguresSupplementRevisedFigure3.pdf]

Anna-Catharina Scholpp - Supplement Figure 4.

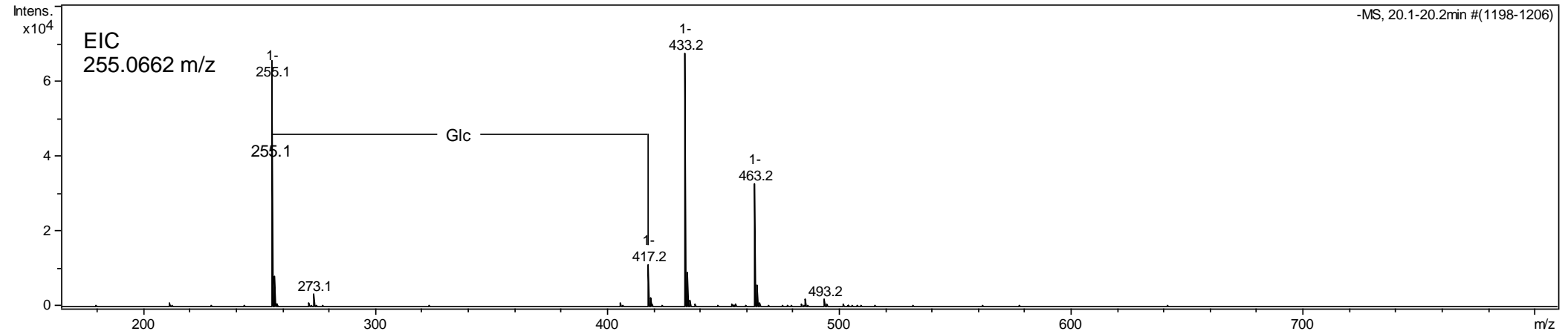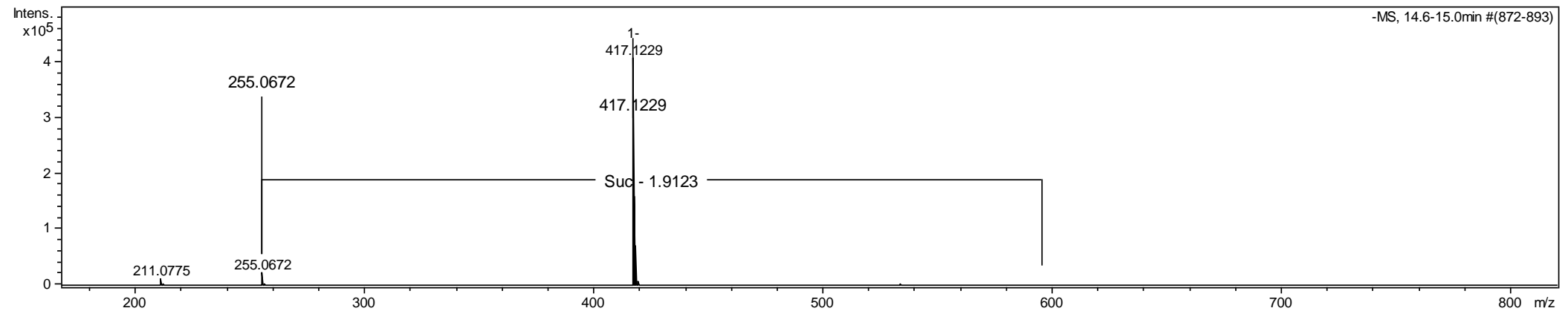

Supplement: Supplementary file 4 [file DataSheet1.zip › FiguresSupplementRevised.pdf/FiguresSupplementRevisedFigure4.pdf]

Anna-Catharina Scholpp - Supplement Figure 5.

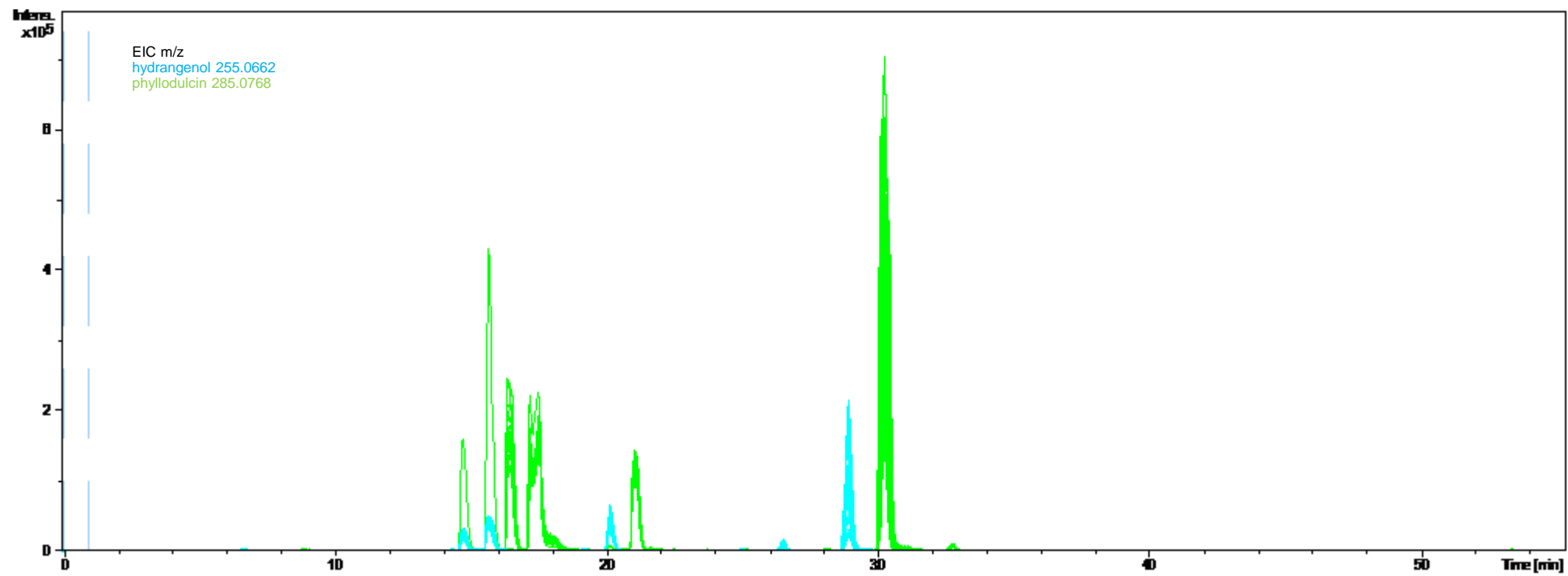

Supplement: Supplementary file 4 [file DataSheet1.zip › FiguresSupplementRevised.pdf/FiguresSupplementRevisedFigure5.pdf]

Anna-Catharina Scholpp - Supplement Figure 6.

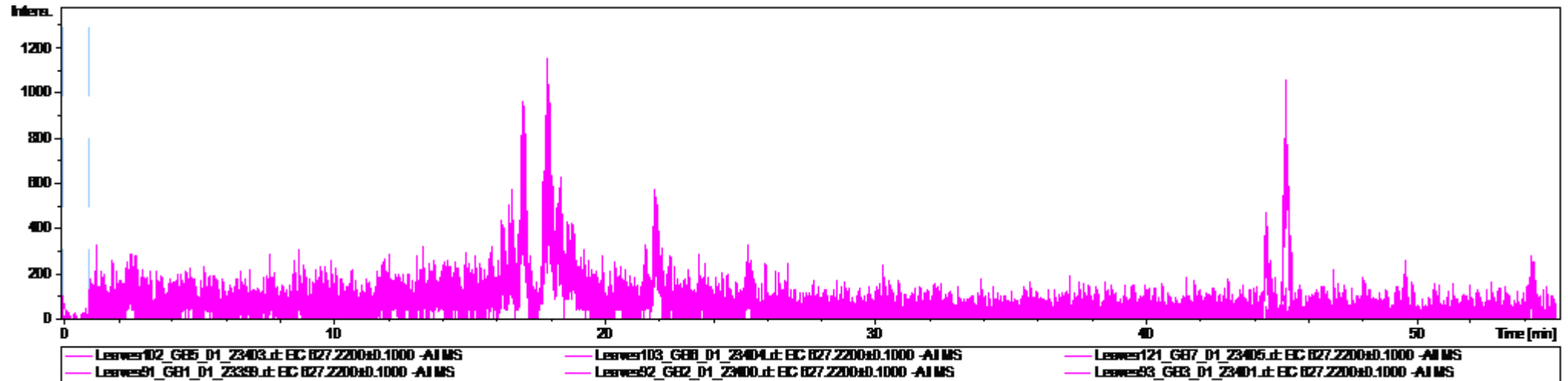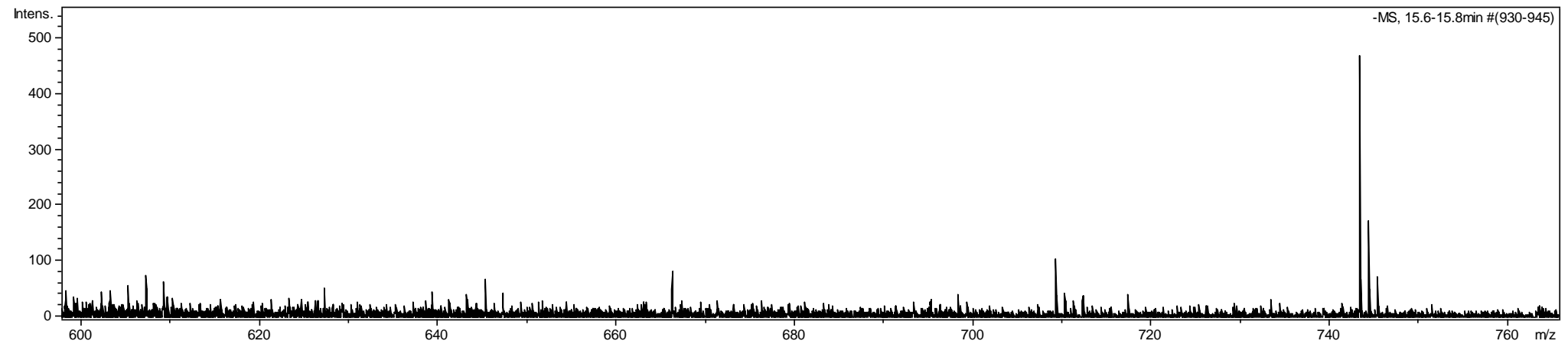

Supplement: Supplementary file 4 [file DataSheet1.zip › FiguresSupplementRevised.pdf/FiguresSupplementRevisedFigure6.pdf]

Anna-Catharina Scholpp - Supplement Figure 7.

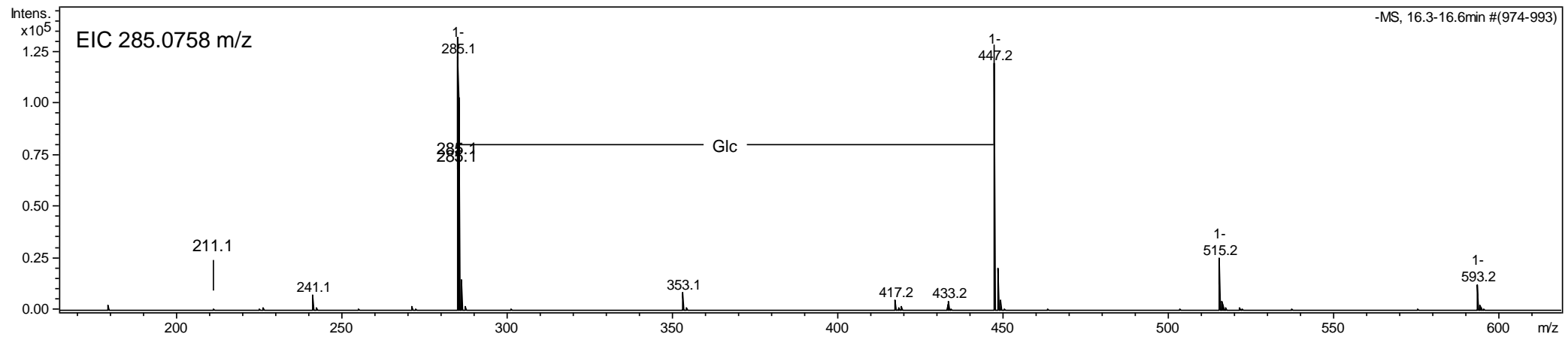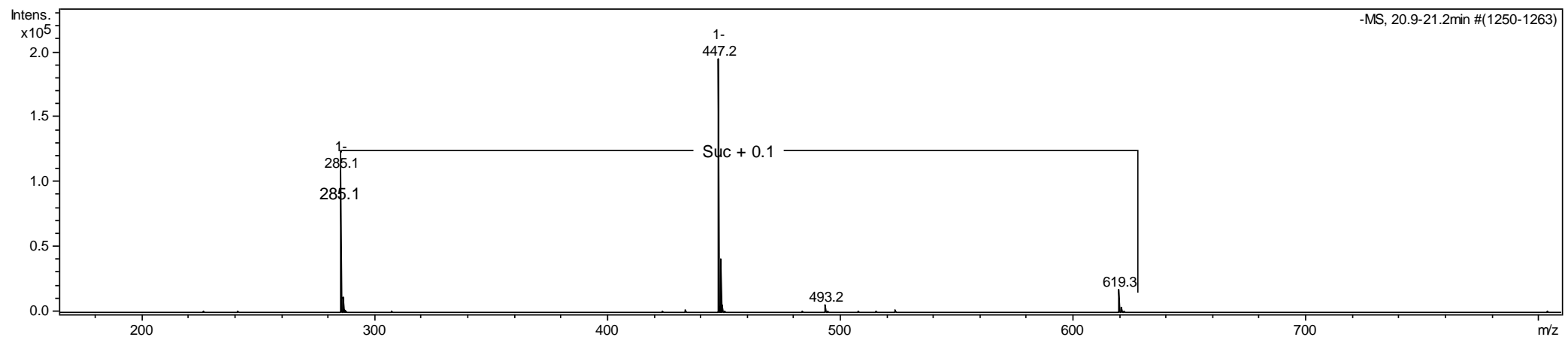

Supplement: Supplementary file 4 [file DataSheet1.zip › FiguresSupplementRevised.pdf/FiguresSupplementRevisedFigure7.pdf]
