# Supplementary material for: Differential gene expression in leaves and roots of Hydrangea serrata treated with aluminium chloride
Source: Front Plant Sci. 2024 Sep 3;15:1412189. doi: 10.3389/fpls.2024.1412189 (PMC11405211; doi:10.3389/fpls.2024.1412189)
Supplement: Supplementary file 5 [file DataSheet2.pdf]

Differential gene expression in leaves and roots of Hydrangea serrata treated with aluminium chloride

Anna-Catharina Scholpp[1], Hanna Marie Schilbert[2], Prisca Viehöver[2], Bernd Weisshaar[2], Michael Beckstette[3], Judith Martha Neumann[1], Hanna Bednarz[1,4] & Karsten Niehaus[1*]

1 Metabolome- and Proteome Research, Faculty of Biology & CeBiTec, Bielefeld University
2 Genetics and Genomics of Plants, Faculty of Biology, CeBiTec, Bielefeld University
3 Center for Biotechnology (CeBiTec), Faculty of Technology, Bielefeld University
4 Medical School OWL, AG 3 Anatomy and Cell Biology

Supplementary Material and Methods

Sample preparation for LC-MS measurement

Hydrangea serrata freeze dried tissue material was analysed for dihydroisocoumarins with focus on relative hydrangenol, phyllodulcin, hydrangeic acid and their glycosidic contents. 200 mg ± 10 mg of each replicate was ground and combined with 500 mg of 1 mm Zirconia beads. 1 mL of ice cold 80 % methanol including naringenin (Carl Roth, Karlsruhe, Germany) as internal standard with final concentration 0.04 mg/mL was added. The mixture was ribolysed 3x6000 rpm 30 sec pause and extracted at 4 °C, overnight. After centrifugation 14600 rpm for 20 min the particle free supernatant was transferred into fresh tubes. To the leave extracts was added 1 mL of n-Hexane. The solution was mixed by inverting 15x and then let separate the solvent phases. The lower methanol phase was transferred into a fresh tube. For further precipitation of still solved impurities, the extracts were stored over night at 4 °C and centrifuged the next day. The clear supernatant was transferred into glass vials with septum (WICOM, Heppenheim, Germany) and used for mass spectrometry measurement.

Table 1. UHPLC-ESI-QToF-MS settings and gradient for dihydroisocoumarin separation

LC parameters

solvents	**A** HPLC grade Water + 0.1 % formate
	**B** HPLC grade Acetonitrile + 0.1 % formate
flow	0.45 mL/min
column oven temperature	40 °C
injection volume	10 µL

ESI-MS parameters

dry gas	8 L/min
nebulizer gas	3 Bar
dry temperature	180 °C
mass detection range	120-1000 m/z
collision cell collision energy	40 eV

Supplementary Results and Figures

Relative DH content in *Hydrangea serrata* roots and leaves. UHPLC-ESI-QToF-MS analysis revealed a tissue specific profile.

Figure 1. TIC (total ion chromatogram) of methanolic *Hydrangea serrata* root extracts in UHPLC-ESI-QToF-MS measurement for dihydroisocoumarin profiling. Turquoise spectra indicate samples from aluminium chloride ($AlCl_3$) treated plants im comparison to the red spectra, which indicate control.

Figure 2. TIC (total ion chromatogram) of methanolic *Hydrangea serrata* leaf extracts in UHPLC-ESI-QToF-MS measurement for dihydroisocoumarin profiling. Blue spectra indicate samples from aluminium chloride ($AlCl_3$) treated plants im comparison to the orange spectra, which indicate control.

Figure 3. Methanolic extracts from aluminium chloride ($AlCl_3$) treated *Hydrangea serrata* roots and control roots in UHPLC-ESI-QToF-MS measurement for dihydroisocoumarin profiling. Hydrangenol, hydrangenol-glycoside, hydrangenol-diglycoside, phyllodulcin and phyllodulcin-glycoside were identified in root extracts but there is no difference in samples from $AlCl_3$ treatment group compared to control. Glycosides and dihydroisocoumarins modified with disaccharide eluate at different retention times. The chromatogram shows the elution profile of the two dihydroisocoumarins assessed as hydrangenol $[M-H+]^-$ 255.06 m/z and $[M-H+]^-$ phyllodulcin 285.16 m/z obtained by extracted ion chromatogram (EIC) over a run of 55 min. Hydrangeic acid and hydrangenol are isobars. Hydrangeic acid should elute at shorter different retention time because it has a carboxylic acid group instead of the closed ring.

Figure 4. Mass spectrum of dihydroisocoumarin assessed as hydrangenol $[M-H+]^-$ 255.0662 m/z and hydrangenol-glycoside 417.12 m/z, as well as hydrangenol-diglycoside 589.20 m/z. Glc indicates modification of the metabolite with a molecule of 162 Da which is the mass of a hexose attached via glycosidic bond. Suc indicates the modification with the disaccharide sucrose.

Figure 5. Methanolic extracts from aluminium chloride ($AlCl_3$) treated *Hydrangea serrata* leaves and control leaves in UHPLC-ESI-QToF-MS measurement for dihydroisocoumarin profiling. Hydrangenol, hydrangenol-glycoside, phyllodulcin, phyllodulcin-glycoside and phyllodulcin-diglycoside were identified. Phyllodulcin is more present in leaf extracts compared to hydrangenol. There is no dihydroisocoumarin relative content difference in $AlCl_3$ treatment compared to control. Glycosides and dihydroisocoumarins modified with disaccharide eluate at different retention times. The chromatogram shows the elution profile of the two dihydroisocoumarins assessed as hydrangenol $[M-H+]^-$ 255.06 m/z and $[M-H+]^-$ phyllodulcin 285.16 m/z obtained by extracted ion chromatogram (EIC) over a run of 55 min.

Figure 6. Extracted ion count chromatogram of dihydroisocoumarin assessed as $[M-H+]^-$ phyllodulcin-diglycoside 627.19 m/z.

Figure 7. Mass spectrum of dihydroisocoumarin assessed as phyllodulcin $[M-H+]^-$ 285.0758 m/z and phyllodulcin-glycoside 447.20 m/z as well as phyllodulcin-diglycoside 627.19 m/z. Glc indicates modification of the metabolite with a molecule of 162 Da which is the mass of a hexose attached via glycosidic bond. to Suc indicates the modification with the disaccharide sucrose. Methanolic extracts from aluminium chloride ($AlCl_3$) treated *Hydrangea serrata* leaves and control leaves in UHPLC-ESI-QToF-MS measurement for dihydroisocoumarin profiling.

Comparison of homology

580,770 contigs were obtained from the Trinity *de novo* assembly. The accession of Trinity IDs encodes the Trinity “gene” and “isoform” information. At the contig level of transcripts, it is not possible to resolve the number of alleles and/or paralogs for one gene without reference genome.

As one example, the level of phylogeny was compared of all contigs (CDS) which were functionally annotated as *pal*. A multiple sequence alignment on cDNA level was made using clustalW (https://www.genome.jp/tools-bin/clustalw). The phylogenetic analysis pipeline by ETE3 on GenomeNet (https://www.genome.jp/tools/ete/) was used to do an alignment and phylogenetic constructions using ETE3 3.1.2 (Huerta-Cepas et al., 2016).

A phylogram was constructed and visualised using fasttree (Price et al., 2010). This phylogram of all fiftyfive contigs which were functionally annotated as *pal* (phenylalanine ammonia lyase) from the *de novo* transcriptome of *H. serrata*. The values at the tree nodes are SH-like for maximum likelihood. The Trinity IDs with the same main number from one gene cluster (c) which were assigned as “isoforms” (i) show small distances in the tree.
